# Mechanism for the Unfolding of the TOP7 Protein in Steered Molecular Dynamics Simulations as Revealed by Mutual Information Analysis

**DOI:** 10.3389/fmolb.2021.696609

**Published:** 2021-09-30

**Authors:** Ognjen Perišić, Willy Wriggers

**Affiliations:** ^1^ Big Blue Genomics, Belgrade, Serbia; ^2^ Department of Mechanical and Aerospace Engineering, Old Dominion University, Norfolk, VA, United States

**Keywords:** molecular dynamics analysis, protein design and engineering, protein unfolding pathway, mutual information (MI), steered molecular dynamics (SMD)

## Abstract

We employed mutual information (MI) analysis to detect motions affecting the mechanical resistance of the human-engineered protein Top7. The results are based on the MI analysis of pair contact correlations measured in steered molecular dynamics (SMD) trajectories and their statistical dependence on global unfolding. This study is the first application of the MI analysis to SMD forced unfolding, and we furnish specific SMD recommendations for the utility of parameters and options in the *TimeScapes* package. The MI analysis provided a global overview of the effect of perturbation on the stability of the protein. We also employed a more conventional trajectory analysis for a detailed description of the mechanical resistance of Top7. Specifically, we investigated 1) the hydropathy of the interactions of structural segments, 2) the H_2_O concentration near residues relevant for unfolding, and 3) the changing hydrogen bonding patterns and main chain dihedral angles. The results show that the application of MI in the study of protein mechanical resistance can be useful for the engineering of more resistant mutants when combined with conventional analysis. We propose a novel mutation design based on the hydropathy of residues that would stabilize the unfolding region by mimicking its more stable symmetry mate. The proposed design process does not involve the introduction of covalent crosslinks, so it has the potential to preserve the conformational space and unfolding pathway of the protein.

## Introduction

**FIGURE 1:**
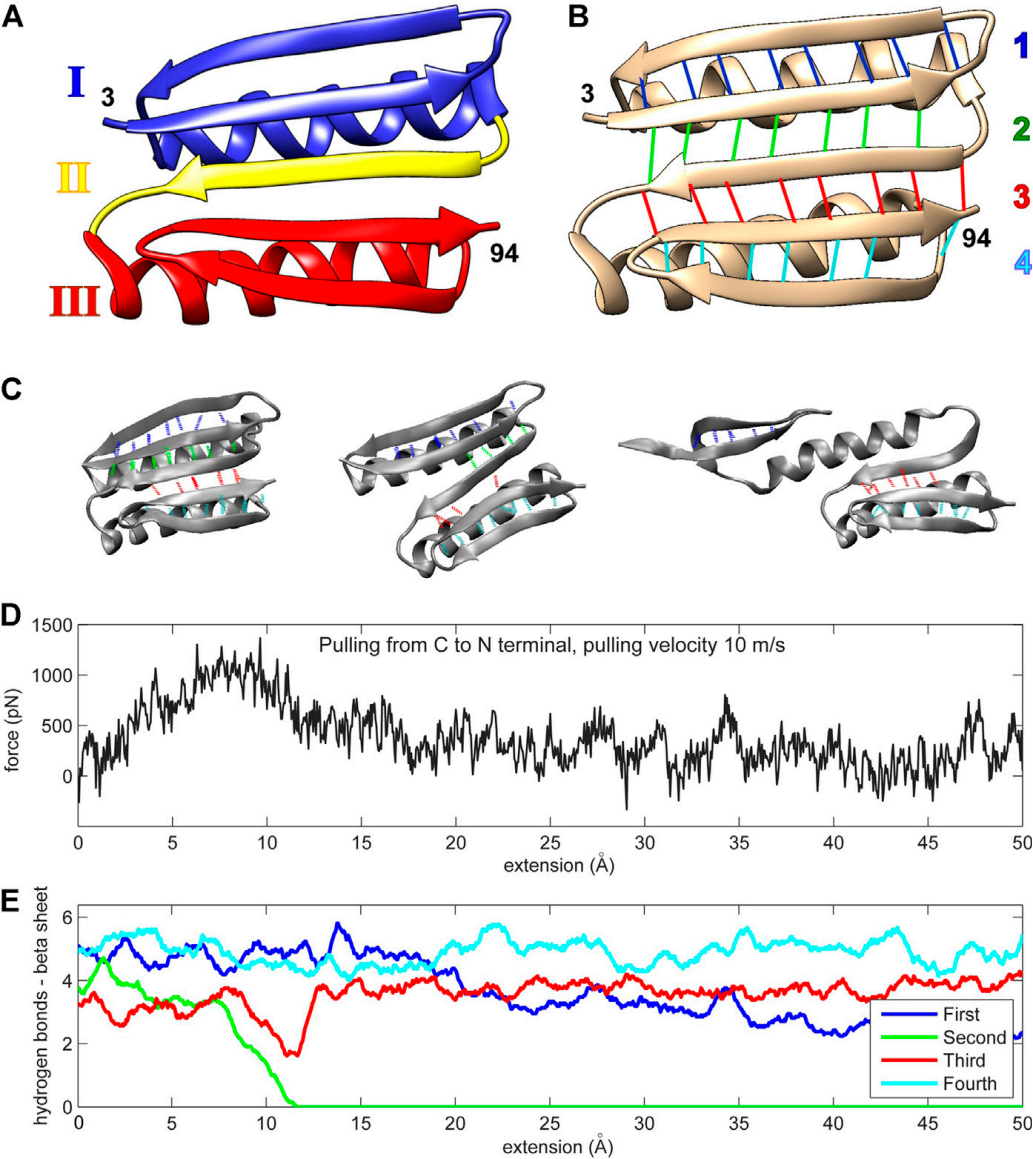
**(A)** The Top7 protein is composed of two alpha helices and a single beta sheet. The structure can be divided into two segments, each comprised of an alpha helix and a part of the beta sheet (regions I and III). A single beta strand (region II) separates these segments. The first and last residues in the protein chain are enumerated (numbers 3 and 94 in PDB ID 1QYS). **(B)** The beta sheet is formed by five strands that are connected by four sets of hydrogen bonds. The first hydrogen bond set is colored blue (connecting two strands with residues 3–12 and 15–25), the second set is colored green (residues 3–12 and 46–55), the third set is colored red (residues 46–55 and 86–94), and the fourth set is colored cyan (residues 76–85 and 86–94). The two alpha helices comprise residues 26–45 and residues 56–75. **(C)** Three protein conformation snapshots during the unfolding. The hydrogen bonds are colored as in **(A,D)** The force-extension curve (pulling velocity 10 m/s). **(E)** Number of hydrogen bonds (see Methods section) during the simulation (pulling velocity 10 m/s). The continuous values are obtained via low-pass filtering (moving average with window width 11 frames, or ± 5 frames about sampling points). Panels **(A,B)** were created with Chimera (Pettersen et al., 2004). Panel **(C)** and all other molecular structure graphics in this work were created with VMD (Humphrey et al., 1996).

Top7 is a human-engineered protein with resistance to unfolding comparable to that encountered in naturally occurring proteins that exhibit mechanical roles (Kuhlman et al., 2003). Its tertiary structure has not yet been encountered in nature (Figure 1). The protein is composed of two alpha helices and one beta sheet. The protein’s uniqueness stems from its beta sheet, which consists of five strands (residues 15–25, 3–12, 46–55, 86–94, and 76–85, as shown from top to bottom in Figure 1B). The N- and C-terminal strands (which are force-bearing in mechanical pulling experiments or simulations) are the second and penultimate strands, respectively (Figure 1B). The middle strand of the beta sheet (also known as region II; Figure 1A) is sequentially distant from these force-bearing strands but connected to them through hydrogen bonds (Figure 1B). The Top7 fold is symmetrical about the central region II and can be divided into two structurally similar segments, each having an alpha helix and two stands of the beta sheet (regions I and III; Figure 1A).

The initial molecular dynamics (MD) simulations (Sharma et al., 2007; Genchev et al., 2009) showed that a fast, simulated perturbation far from equilibrium can be used as a guideline in the rational design of Top7. During the simulated pulling, that is, steered MD (SMD), the second group of hydrogen bonds weakens and disappears, which correlates with the drop in resistance force (Figures 1C–E). This process is almost identical for every pulling regime examined (1, 5, or 10 m/s pulling) and pulling direction (from N or C terminus), except for the fastest pulling (100 m/s) when the second and third groups break together. The pulling regimes were far from equilibrium—seven to eight orders of magnitude faster than the real-world constant-velocity pulling experiments (Rief et al., 1997; Carrion-Vazquez et al., 2003; Eckels et al., 2019).

Mechanical resistance of Top7 is reflected in the behavior of its beta sheet hydrogen bonds. The protein unfolds into three parts: The first part is a fragment of the beta sheet belonging to structural region I; the second part is the alpha helix I from region I; and the third part mainly comprises region III, which keeps its overall configuration largely intact and stays loosely bound to helix I (Sharma et al., 2007). The beta sheet is composed of four sets of hydrogen bonds (Figure 1B). The protein’s forced unfolding is mainly reflected in the drop in the number of hydrogen bonds from the second set that connects the central beta strand (region II) to the force-bearing beta strand of region I. In the initial work (Sharma et al., 2007; Genchev et al., 2009), a disulfide bond introduced in the protein’s beta sheet forced the protein to follow the unfolding pathway of high resistance. Covalent cross-linking is evidently a rather intrusive manipulation that alters the unfolding pathway by introducing (non-native) spatial constraints. In the present work, we used a different approach and sought possible mutants that strengthen non-covalent contacts, leaving the polypeptide chain intact.

Our new design paradigm for enhancing mechanical stability is based on a statistical analysis of residues important for the global unfolding process, as well as on an analysis of the biophysical and biochemical properties of SMD trajectories, which is different from the cross-linking strategy originally applied by Sharma et al. (2007). The present work is an extension of previously proposed strategies (Wriggers et al., 2009; Shaw et al., 2010; Kovacs and Wriggers, 2016; Kovacs et al., 2017) aimed at detecting functionally relevant mechanisms in proteins. The new approach efficiently matches fast local movements (secondary or tertiary structure rearrangements) with slow global protein behavior (characterized by an activity function or order parameter). Statistical dependence using mutual information (MI) generates a spatial heat map that reveals functionally relevant protein segments from the time series. The MI analysis reveals only statistically correlated residues and does not offer a physical interpretation of the protein’s behavior during stretching. The physical properties of those residues, such as their water molecule binding and hydrogen bonding, can offer insights into the true mechanism of the Top7’s asymmetrical response to the external stimulus.

## Methods

### Steered Molecular Dynamics Simulations of Top7

Recent advances in parallel computing have made MD simulations accessible to a wide range of researchers who can now regularly perform long simulations of very large biomolecular complexes, such as viral capsids (Arkhipov et al., 2006; Perilla and Schulten, 2017; Tarasova et al., 2017), full viral particles (Freddolino et al., 2006; Wang et al., 2013), ribosomes (Sanbonmatsu, 2012), or chromatin fibers (Sanbonmatsu, 2019). The success of such studies in protein folding, drug design, or structure optimization primarily relies on the numerical power of modern architectures and on the accuracy of algorithms and force fields. Such technical challenges have been addressed in earlier works (Sharma et al., 2007; Genchev et al., 2009) on the force-induced unfolding of the Top7 protein. Figure 1 provides a review of the existing SMD simulations (Sharma et al., 2007; Genchev et al., 2009) we used in this study.

The Top7 protein equilibration and SMD constant velocity stretching simulations were performed at 310 K in an explicit water solvent box with periodic boundary conditions large enough for a spring extension of up to 50 Å (length 127 Å, width 52 Å, height 59 Å). The SMD spring constant was 10 kcal/mol/Å^2^. The protein–solvent system contained 39,121 atoms. The spring velocities used in the SMD simulations were between 1 and 100 m/s (in this paper, we used simulations based on 1 and 10 m/s velocities). The model was prepared with VMD (Humphrey et al., 1996), and MD simulations were performed with NAMD (Phillips et al., 2005) by using par_all27_prot_lipid (CHARMM22/27) parameters and topologies. During the 1 ns equilibration simulations, the protein was stable within a 2 Å root-mean-square deviation (RMSD) from the initial PDB structure 1QYS. The equilibrated structures were the starting structures used in the SMD simulations. The SMD simulation length (integration time step: 1 fs) was determined by the spring velocity; for example, at 50 Å extension and 10 m/s velocity, the SMD part of the simulation was 0.5 ns long, and at 25 Å extension and 1 m/s velocity, the SMD part of the simulation was 2.5 ns long.

### Mutual Information Analysis of MD Trajectories Using *TimeScapes*

The analysis of the SMD simulation trajectories presents a challenge. Functionally important effects are often difficult to detect visually (Humphrey et al., 1996; Pettersen et al., 2004). Therefore, we visualized unfolding events locally by using information theory—by transforming highly resolved SMD time series into spatial images or heat maps that characterize the importance of specific amino acid residues for the unfolding. The statistical analysis described in the following was carried out with the Python-based *TimeScapes* package (Wriggers et al., 2009; Kovacs and Wriggers, 2016) disseminated at http://timescapes.biomachina.org.

Specifically, in the present work, we used the tagging.py tool, which represents an MD trajectory as a time series of pairwise side-chain distances $X_{ij}\left(t\right)$ , where each amino acid (denoted here by *i* or *j*) is represented by an atom from its side chain. Thus, the $X_{ij}\left(t\right)$ measures fast local rearrangements of the protein configuration. The coarse graining into side-chain interactions makes the problem more tractable (the computational complexity scales with the square of the number of residues but not with the square of the number of atoms). We used default parameters, that is, neighbor contact exclusion in the coarse model (excl = 1), distance cutoff set to infinity (i.e., no cutoff), and the default coarse-graining function (mod_pwk_side).

When a folding or unfolding protein transitions between relatively quiescent “low activity” basins to other such basins as a function of simulation time, it experiences “high activity” bursts during the transition (Wriggers et al., 2009). In the following, we statistically related the rate of change of the $X_{ij}\left(t\right)$ to a function that measures the activity during such folding or unfolding events. Several types of activity functions a(t) supported in *TimeScapes* (Wriggers et al., 2009; Kovacs and Wriggers, 2016) provide different imaging modalities for visualization. Because of its robustness and demonstrated utility in characterizing folding simulations (Kovacs and Wriggers, 2016), we used the RMS fluctuation (RMSF) of Cartesian coordinates in a Gaussian-weighted sliding window of length *δ*, which was computed with the agility.py tool.

If I(x,y) denotes a measure of the statistical dependence of two discrete random variables *x* and *y*, it can then be used to express the measure $R_{X,a}\left(i,j\right)$ of the dependence between local changes of $X_{ij}\left(t\right)$ and the global activity function a(t) as (Kovacs and Wriggers, 2016):

$$R_{X,a}\left(i,j\right)=I\left(\left|\frac{dX_{ij}\left(t\right)}{dt}\right|,a\left(t\right)\right)$$ (1)

**FIGURE 2:**
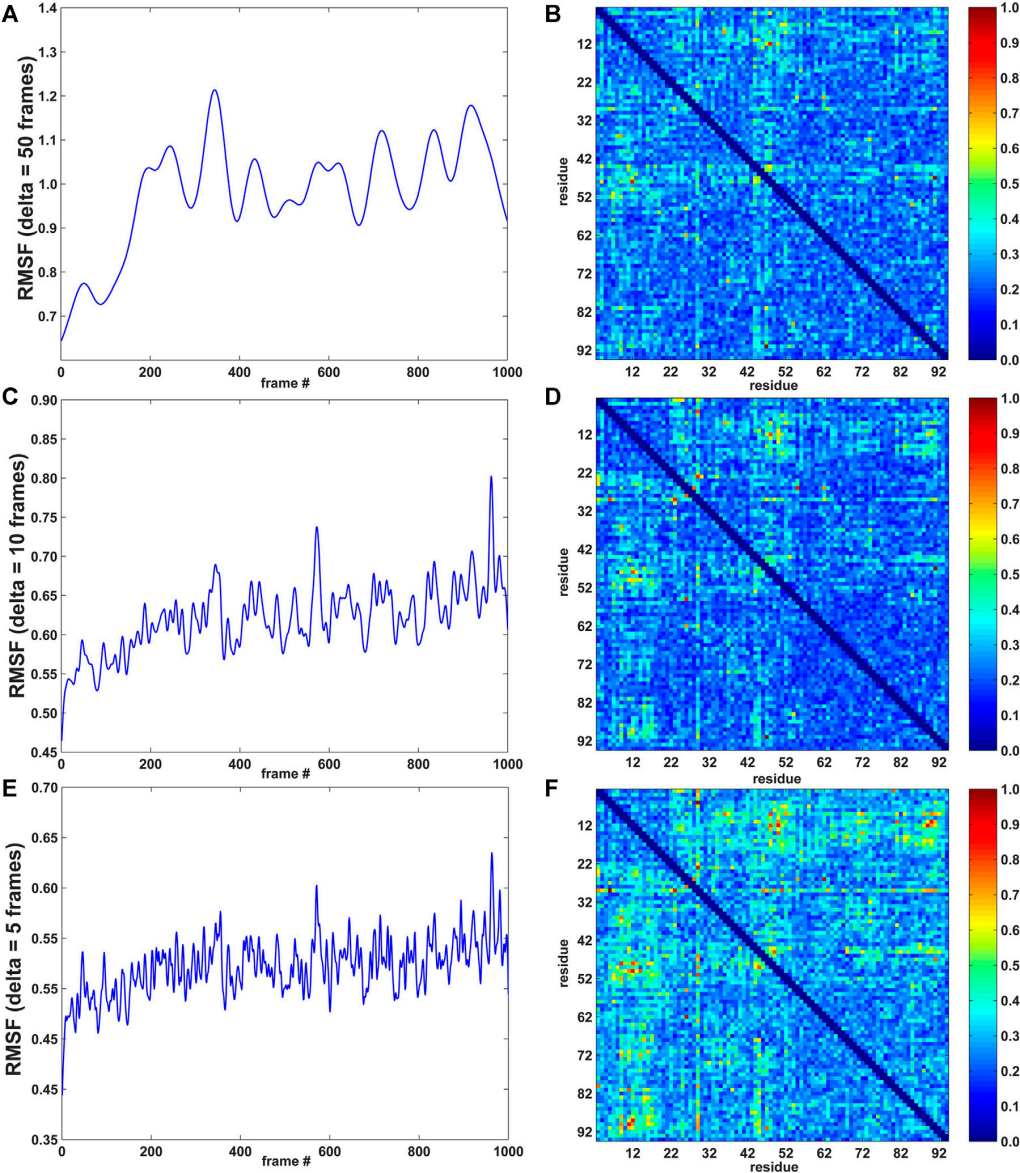
RMSF curves (left; arbitrary units) and pairwise residue MI matrices (right; normalized to a range of [0, 1]) for three values of *δ* (window width: 50, 10, and 5 frames; 1 frame = 0.5 ps for the 10 m/s SMD velocity; see Methods section). **(A)** RMSF for *δ* = 50 frames. **(B)** MI pairwise matrix for *δ* = 50 frames. **(C)** RMSF for *δ* = 10 frames. **(D)** MI pairwise matrix for *δ* = 10 frames. **(E)** RMSF for *δ* = 5 frames. **(F)** MI pairwise matrix for *δ* = 5 frames.

I(x,y) was initially expressed as a linear Pearson cross-correlation (Wriggers et al., 2009), but we recently adopted MI, which is more robust with respect to the choice of activity function, and it provides results even in fast-folding cases, where the earlier cross-correlation failed to detect any significant contacts (Kovacs and Wriggers, 2016). The coefficients $R_{X,a}\left(i,j\right)$ can then be used to rank the local residue pairs (*i*,*j*) by the statistical dependence of their fast motion on the slow, global activity a(t) . The $R_{X,a}\left(i,j\right)$ forms a heat map that can be plotted as a 2D matrix (Figure 2, right).

Eq. 1 was originally developed for non-negative activities (Kovacs and Wriggers, 2016), but tagging.py also supports a variant of the approach, where an external raw time series is used as an order parameter instead of an activity function; furthermore, instead of the absolute rate of change, the direct dependence on (signed) pairwise distances is computed:

$$R_{X,a}\left(i,j\right)=I\left(X_{ij}\left(t\right),a\left(t\right)\right)$$ (2)

Here, a(t) may be signed and denotes an external order parameter that characterizes the global state of the system. The approach in Eq. 2 was historically used in the analysis of the first millisecond MD simulation (Supplementary Figure S9 of Shaw et al., 2010), where a(t) denotes the membership of specific conformational clusters in Supplementary Table S2 of Shaw et al. (2010). In the present work, we focused on force-induced unfolding, similar to that in a(t) in Eq. 2, where we used the RMSD from the initial conformation (computed by VMD; Humphrey et al., 1996), in addition to the RMSF.

The Python-based package *TimeScapes* was recently enhanced with additional C++ code for the balanced approach to adaptive probability density estimation (BADE) (Kovacs et al., 2017): Python programs that support MI, such as tagging.py, execute the BADE program (Wriggers, 2017) and use the resulting probability densities for the MI calculations in Eqs. 1, 2.

### Biomolecular Trajectory Analysis

For the hydropathy analysis, the protein was divided into structural sub-segments based on visual inspection of how secondary structure elements move during the perturbation. For specific pairs of such sub-segments, the degree of hydrophobicity was computed for contact and non-contact residues by averaging their Black and Mould (Black and Mould, 1991) or Kyte and Doolittle (Kyte and Doolittle, 2003) hydropathy scores. A residue was defined to be in contact if any of its atoms were within 4.5 Å of the atoms from a neighboring segment. The contact residues were extracted using our in-house developed programs (Perišić, 2018; Perišić, 2020).

The number of hydrogen bonds, water molecule concentration, and angles between residue triplets were measured with VMD (Humphrey et al., 1996) by using Tcl/Tk scripts, which were developed specifically for this purpose. The hydrogen bonds were selected with the VMD list command *set hbcount1* [*llength* [*lindex [measure hbonds 3.5 45 $sel11 $sel12] 0]]*. The command selects all hydrogen bonds within a 3.5 Å cutoff distance, whereas the angle formed by the donor, hydrogen, and acceptor must be within a tolerance of 45° from the expected 180° angle. The script passes through every simulation frame, counts the number of hydrogen bonds for each selection, and stores them in text files. Similarly, the script that counts the number of water molecules around the desired residues uses the VMD atom selection language. For example, water molecules near residue 15 would be counted with *set sel_wm_around_15* [*atomselect top* “*water and name OH2 and within $radius of protein and resid 15*”. The script passes through every simulation frame and saves the number of water molecules with the function calls *set wmcount_around_15 [$sel_wm_around_15 num] and puts $outfile_15* “*$wmcount_around_15*”. Finally, angles between residue triplets (between corresponding Cα atoms) were calculated using elementary trigonometry and saved in text files for further scripting analysis, similar to the above-given procedures for hydrogen bonds and water molecules.

## Results

Protein folding is a relatively slow equilibrium process guided by entropy, whereas forced unfolding is usually far from equilibrium and characterized by the sudden breaking of noncovalent bonds. In SMD, a sudden disruption of a hydrogen bond is characterized by a burst of high-amplitude fluctuations, which means that bond breakage should be followed by high-frequency fluctuations of the corresponding residues. The raw time series, on the other hand, should move in synchrony with slow, large-amplitude global movements that correspond to mechanically more stable structural segments. In the following heat map analysis, we used the rate of change of pairwise distance time series (Eq. 1) to detect the most important residues for destabilizing the structure and raw time series to identify the globally stable rigid segments (Eq. 2). The statistical analysis is followed by more traditional analysis of biophysical and biochemical properties that further elucidate the unfolding mechanism.

### Rate of Change Mutual Information Analysis Based on the Activity Function

As described in the Methods section, the agility.py script of the *TimeScapes* package was used to extract the RMSF in a Gaussian-weighted sliding window. An important parameter in estimating the RMSF is the length *δ* of the window. The best practices (Wriggers, 2017) suggest starting with 5% of the total number of trajectory frames as the *δ* length and adjusting this value if indicated. In our case, with a length of 1,000 frames, 5% corresponds to 50 frames. To investigate the robustness of the analysis, we also calculated the RMSF with *δ* = 10 and 5 frames. The MI values did not significantly depend on the window length *δ* in earlier equilibrium MD simulations of native state dynamics (Kovacs and Wriggers, 2016), but we would not expect this time invariance in the presence of a significant time-dependent perturbation, such as in the SMD.

In Figure 2, the left part shows the RMSF activity functions obtained with *δ* = 50, 10, and 5 frames (1 frame = 0.5 ps for the 10 m/s SMD velocity, see Methods section), and the right part shows the corresponding normalized pairwise residue MI heat maps based on the absolute derivatives of the residue–residue distance time series (Eq. 1). Figure 2 suggests that the timescale imposed by the pulling regime in (non-equilibrium) SMD has a modest effect on time-dependent statistics, whereas MI values did not significantly depend on the window length *δ* in conventional, equilibrium MD simulations (Supplementary Figure S4 of Kovacs and Wriggers, 2016).

**FIGURE 3:**
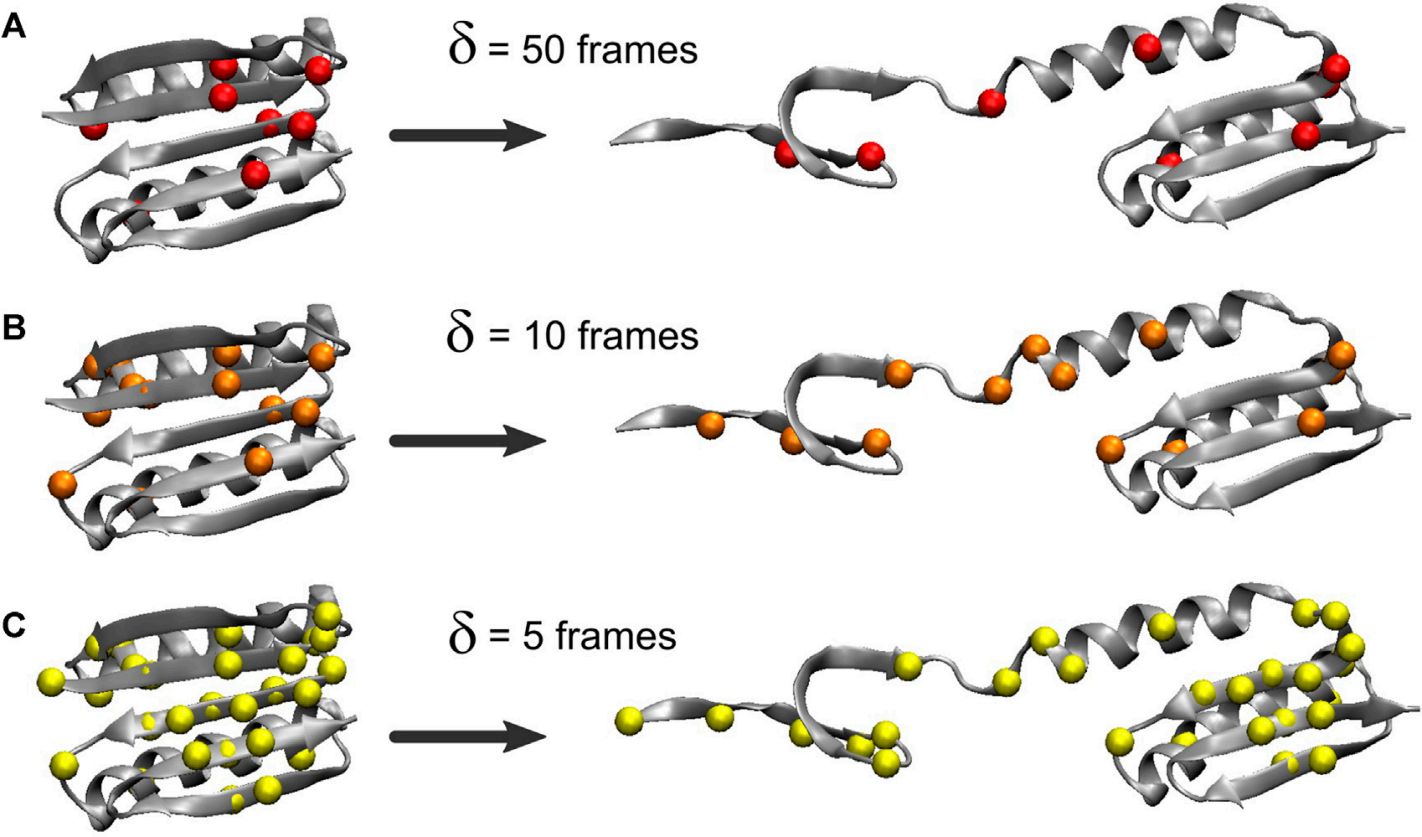
Residues highlighted by the MI analysis of the constant velocity unfolding trajectory. The shown residues were taken from the residue pairs extracted from the activity matrix when the corresponding normalized matrix elements were greater than 0.7. The residues are depicted by their Cα atoms and correspond to matrix elements in Figure 2 (right). The visualization is based on the analysis with the RMSF smoothing windows of *δ* = 50 **(A)**, 10 **(B)**, and 5 **(C)** frames used in Figure 2 *δ* = 50 emphasizes residues 9, 12, 26, 36, 47, 48, 62, and 91, *δ* = 10 emphasizes residues 6, 9, 12, 23, 26, 28, 29, 36, 47, 48, 55, 62, and 91, and *δ* = 5 emphasizes residues 3, 6, 9, 11, 12, 14, 23, 26, 28, 29, 36, 45–52, 55, 62, 72, 80, 81, and 89–91.

The heat map matrices in Figure 2 (right) generally exhibit a banded structure with high MI values for residues 3–22 (the first two beta strands from region I) and the central strand region II but lower values in region III. However, the maps are relatively noisy. For an improved visualization of the results in 3D, Figure 3 depicts residues extracted from the 2D heat maps when corresponding normalized matrix elements were greater than 0.7. With *δ* = 50 frames, the MI analysis emphasizes residue pairs 9/36, 12/48, 26/62, and 47/91. With *δ* = 10 frames, the procedure yields residue pairs 6/29, 9/36, 12/48, 23/29, 26/28, 26/55, 26/62, and 47/91. With *δ* = 5 frames, residue pairs 3/29, 3/80, 6/29, 9/36, 9/48, 9/52, 9/72, 9/90, 11/50, 11/81, 11/90, 12/48, 12/50, 12/89, 12/90, 14/50, 14/72, 23/29, 26/28, 26/55, 26/62, 29/46, 29/49, 29/51, 29/80, 29/90, 29/91, 45/49, 47/91 are selected. Although the highlighted residues generally agree in their location, a sharper RMSF curve provides an opportunity for more pairwise interactions to exhibit a high statistical dependence on the RMSF (the residue selection expanded with decreasing width/duration *δ*).

The analysis primarily highlights residues from the beta sheet belonging to regions I and II (in particular, residues 9 and 12) and, only to a lesser extent, residues in the alpha helices (mostly from region I). For *δ* = 50 and *δ* = 10 frames, Figure 3 emphasizes residues from the structural regions I and II. Those residues are, surprisingly, concentrated far from the site where Sharma et al. (2007) introduced the disulfide bridge (residues 3 and 51) to force the protein to unfold via the third set of beta sheet hydrogen bonds (Figure 1). The analyses with *δ* = 50 and *δ* = 10 frames omit residues 3 and 51, and only the analysis with *δ* = 5 highlights those two among 27 other residues that emerge from the noise in the heat map (Figure 2F).

### Direct Mutual Information Analysis Based on Raw Time Series

**FIGURE 4:**
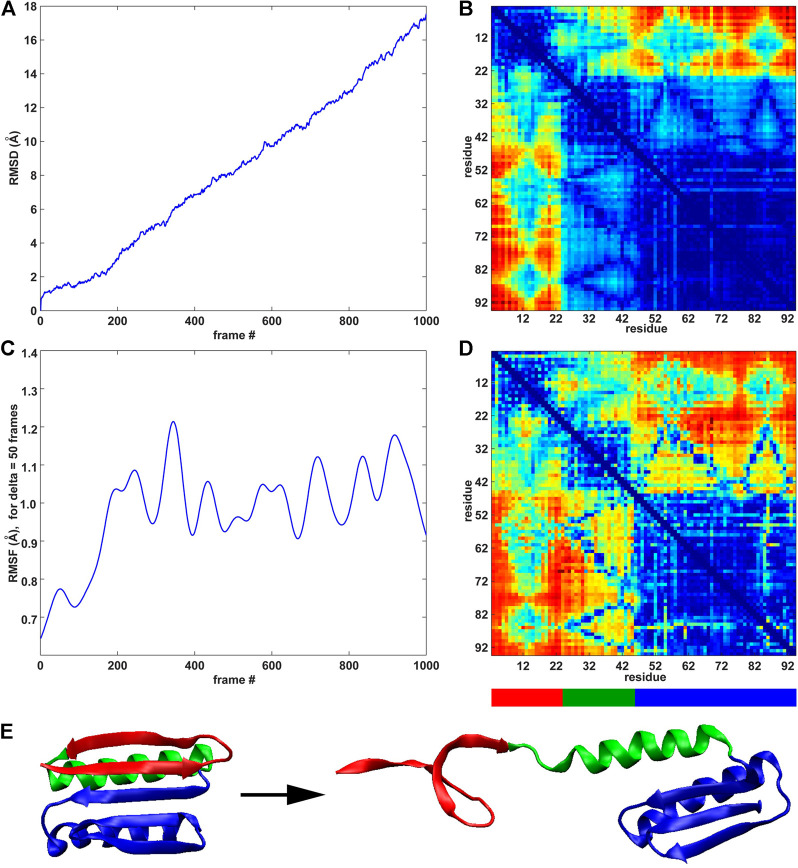
MI analyses of the constant velocity unfolding trajectory against RMSD order parameter and RMSF activity function. **(A)** RMSD curve (see text). **(B)** Pairwise residue matrix created by direct MI against RMSD (color scale as in Figure 2, right). **(C)** RMSF curve (Figure 2A). **(D)** Pairwise residue matrix created by direct MI against RMSF (color scale as in Figure 2, right). **(E)** Cartoon representation of the protein with colored segments emphasized by the MI analysis depicted in **(B,D)**. The red segment (residues 3–22) is the most active (see the highly active band in panel **(B)**), showing the activity of residues 3–22 against all other residues. The green segment (residues 23–44) is less active than the red segment (see panel **(B)**), whereas the blue segment (residues 45–94) remains stable (see text for details).

The previous rate of change analysis (Eq. 1) was originally developed for equilibrium simulations where activity functions, such as the RMSF, that characterize one or more functionally relevant changes in the system can be learned (Kovacs and Wriggers, 2016). However, in the case of the SMD trajectories in this paper, the system undergoes a directional change due to the force-induced unfolding. The well-defined directional perturbation provides a natural order parameter that can be used to directly determine the importance of interactions for the structural change by using the raw time series for the analysis (Eq. 2). We used the RMSD and the RMSF (*δ* = 50 frames) curves for the direct MI analysis of the raw pairwise residue distance time series because these curves characterize the deformation of the system (Figure 4A) and the saturation of the internal changes (Figure 4C).

As was the case in Figure 2 (right), where an identical color scale was used, the results in Figures 4B,D show a heat map in pairwise residue space. However, the results in Figure 4 show a much more distinct block pattern that can be readily interpreted in Figure 4E. The raw time series analyses in Figures 4B,D reveal three structurally distinct segments (as judged by the blue squares on the diagonal, which represent low intra-segment values): 1) The union of regions II and III (blue in Figures 4E) helix I from region I (green in Figures 4E) two beta strands from region I (red in Figure 4E). We noted that low MI values do not directly indicate the absence of internal motion in the three segments of Figure 4E and that the motion (e.g., due to thermal fluctuations) is not statistically correlated with the stretching (RMSD) or global activity (RMSF). In this sense, the MI analysis allows us to mask out any unrelated (non-functional) motion. While both analyses highlight the same segments, the RMSD-based analysis is more selective. As the simulated pulling progresses, the two “red” strands alter their interactions with the neighboring helix I and regions II/III (as evidenced by high inter-segment MI values). Helix I is internally stable, and interactions of helix I with regions II/III change with overall activity (RMSF) and are detected by the statistical analysis, but these interactions do not correlate directly with the stretching (RMSD) itself (as is the case in the interactions of helix I with the two “red” strands).

The above heat map analysis is relatively non-specific, but it allows us to focus on key regions that we explored further in the following section. Figure 4 shows that residues 3–22 (two beta strands from region I) are most active during unfolding. This result agrees with the banded structure of the pairwise matrices in Figure 2, which exhibit high MI values for these residues. Figure 3 also shows that many significant residues (in particular, residues 9 and 12 and their structural neighbors) belong to the interface between regions I and II. However, we still needed a deeper knowledge of the system, as MI alone is not sufficient to provide a complete design strategy for engineering a stable structure. To predict a mutation that mechanically stabilizes the protein, we augmented the heat map analysis with a more conventional trajectory analysis.

### Hydropathy, Solvent Exposure, and Hydrogen Bonding

The above statistical analysis showed that the pairwise residue activity is structurally separated and asymmetric despite the tertiary similarity of regions I and III (Figure 1). Two beta strands from region I (residues 3–22) are much more active and eager to unfold than those from region III (Figure 4). As we were interested in discovering the mechanism responsible for the asymmetric response of the protein to the external stimulus, we decided to examine the hydrophobic properties, solvent exposure, and hydrogen bonding in more detail.

**FIGURE 5:**
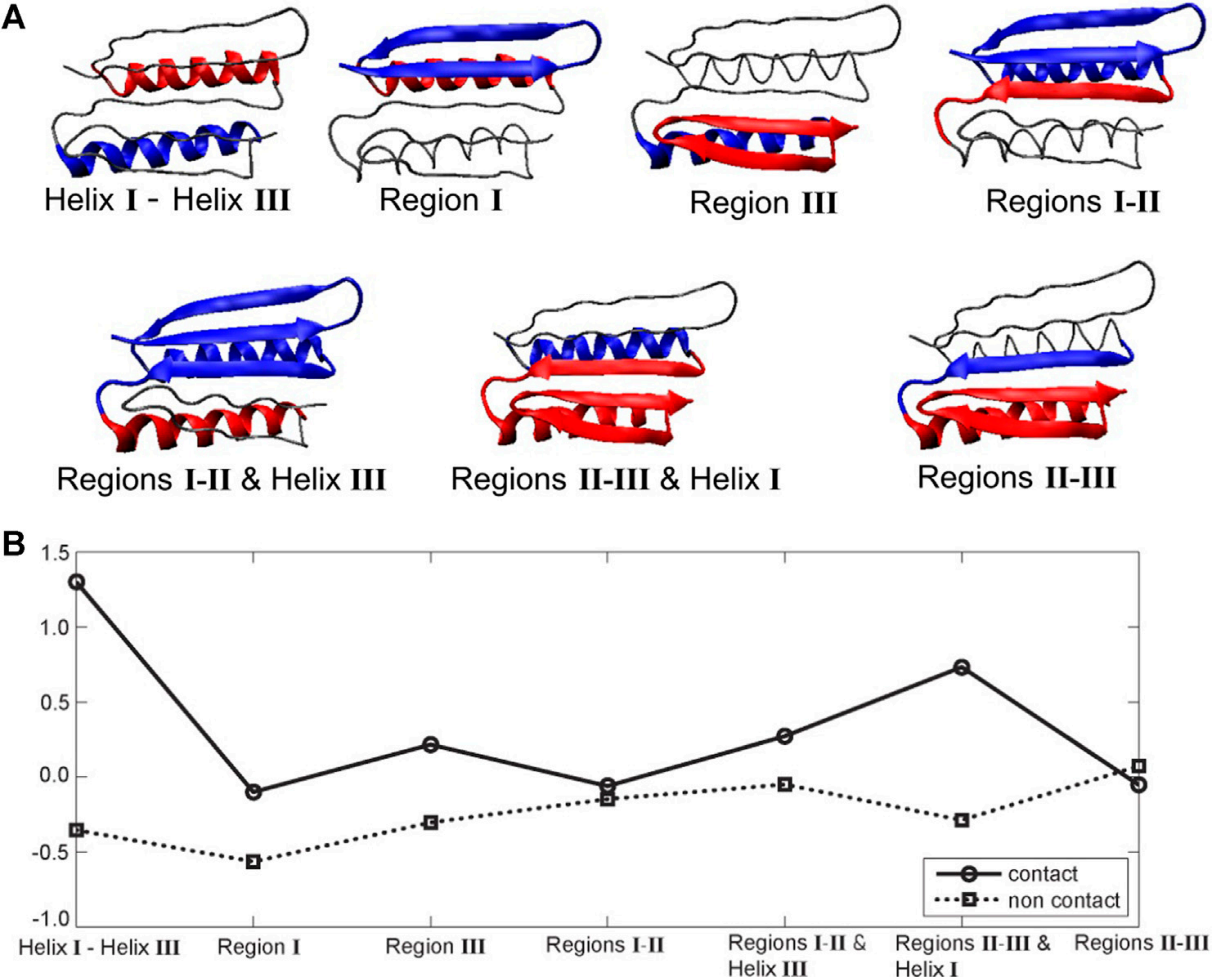
Top7 hydropathy analysis. **(A)** Structural sub-segments (the backbone is represented as a thin line). Each segment is divided into two constituent parts (blue and red). The partitioning was performed heuristically to account for the known functionally important regions that encompass secondary structure elements. **(B)** Corresponding Kyte and Doolittle hydropathy plot (Kyte and Doolittle, 2003; residue specific hydropathy scores averaged over the constituent parts; see Methods section). Negative and positive values indicate the degree of hydrophilicity and hydrophobicity, respectively. Average Black and Mould scores (Black and Mould, 1991; based on non-negative scores that measure only hydrophobic propensity) were also computed but are not shown here because they closely resemble the Kyte and Doolittle contact plot.

The hydrophobic effect is a major driving force in the folding of globular proteins in aqueous solution. To assess the role of specific segments in the Top7’s resistance to pulling, we examined their hydropathy scores (i.e., mutual hydrophobic and hydrophilic propensities) as a potential source of the structural resistance to the external force, as originally suggested by Sharma et al. (2007). Figure 5 shows seven pairs of structural sub-segments and their corresponding average (Kyte and Doolittle, 2003) hydropathy scores (see Methods section), which we related to the MI results.

The largest difference between contact and non-contact hydropathy was observed in the segment composed of helices I and III (Figure 5). This finding means that it should experience the largest change in entropy upon exposing contact residues to the environment. As shown in Figures 1C, 4E, these helices remain attached to each other throughout the pulling process. The observed mechanical resilience would certainly be helped by the pronounced hydrophobic interactions. Furthermore, the segment composed of regions II/III and helix I also has a significant difference between contact and non-contact hydropathy. This effect should also make it stable, which agrees with the green/blue independence from stretching in Figure 4B and the conserved interface in Figures 1C, 4E. Region I has negative contact and non-contact scores. This result indicates that region I overall prefers a water environment over being buried, consistent with the relative ease of its unfolding in Figure 4. Region III is comparable to the symmetry-related region I, but its contact residues have a slight positive score (hydrophobicity) instead. The segment composed of regions I and II and helix III is similar. For these segments, a hydrophobic driving force is present but perhaps not quite as strong as expected. Finally, in regions II and III, as well as regions I and II, we do not observe a significant hydropathy. In fact, the only unexpected result of this analysis was obtained for regions II and III. Based on the stability of the union of regions II and III in Figure 4, we would have predicted a hydrophobic driving force, but none is found, possibly because the strong and stabilizing helix I and III interactions were not included in the scores of regions II and III.

These stability patterns mostly agree with the above MI analysis of the far from equilibrium SMD perturbations and thus suggest that hydrophobicity could play a role in the mechanical resistance to fast pulling of the Top7 protein. However, we must also note that hydrophobicity is an equilibrium property, whereas our simulations are six orders of magnitude faster than those in AFM experiments. Furthermore, similar to the case with the above heat map analysis, the results by themselves are rather global in nature and do not reveal a specific local mechanism. To find additional details, we then analyzed the behavior of specific residues involved in the formation of the protein’s beta sheet and its water binding and hydrogen bonding patterns (Figure 1).

**FIGURE 6:**
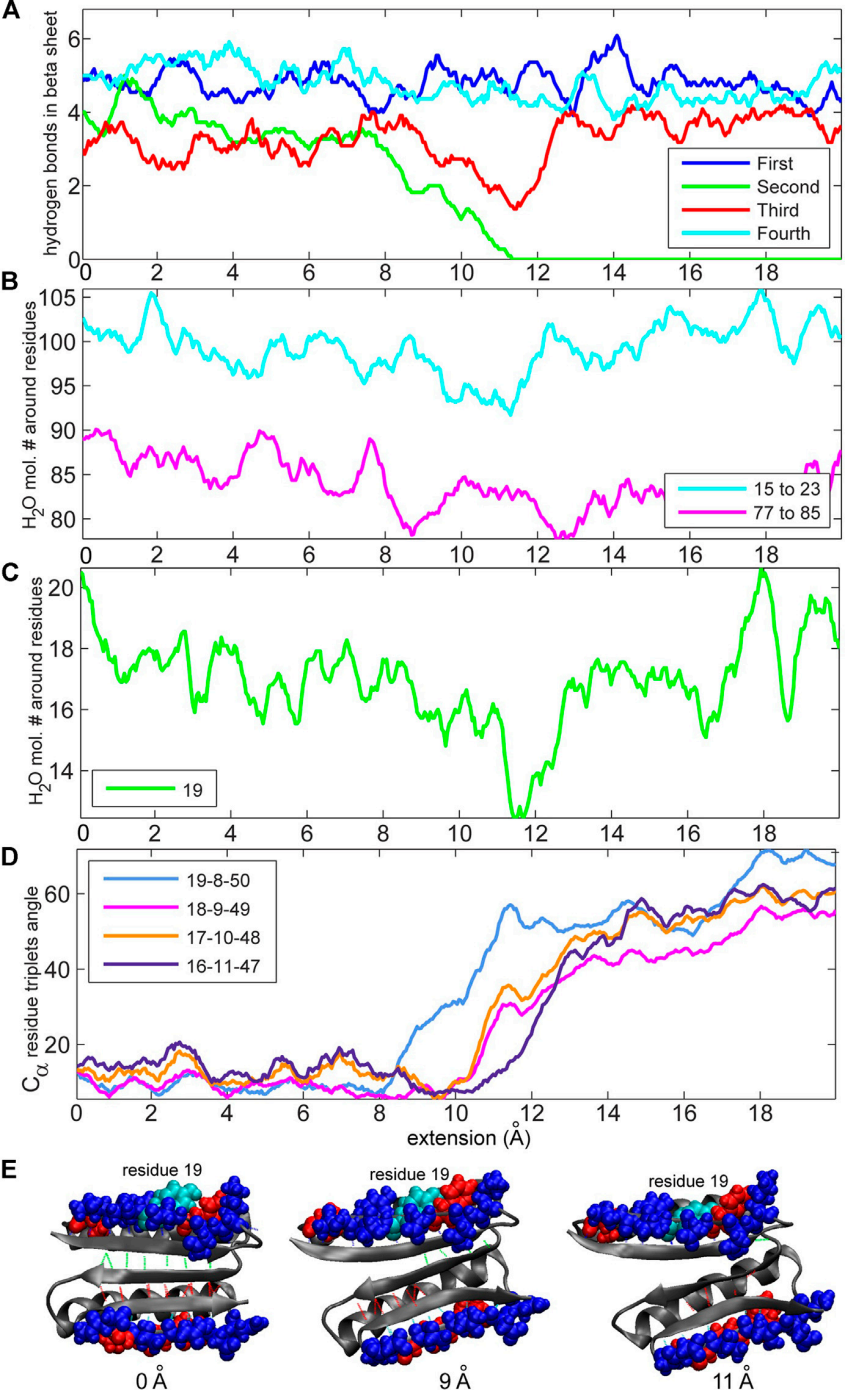
Analyses of the numbers of hydrogen bonds vs. the concentration of water molecules and residue triplet angles (see Methods section) for the 10 m/s trajectory. **(A)** Number of hydrogen bonds during the simulation. The values are obtained through low-pass filtering (moving average with window width 11 frames, or ± 5 frames about sampling points). **(B)** Numbers of water molecules near residues 15–23 and 77–85. **(C)** Number of water molecules near residue 19. **(D)** Residue triplet angles for residue triplets 19–8–50, 18–9–49, 17–10–48, and 16–11–47 (triplet 19–8–50 is discussed in the text; the others are shown for comparison purposes). **(E)** Structure of the protein during pulling. Residue 19 (tyrosine is hydrophobic with a polar side chain) is colored cyan. Hydrophobic residues (15–23 and 77–85) are colored red, and hydrophilic residues are colored blue. Hydrophobic and hydrophilic residues in Figures 6, 8 are displayed with VMD (Humphrey et al., 1996) by using the built-in atom selection language.

**FIGURE 7:**
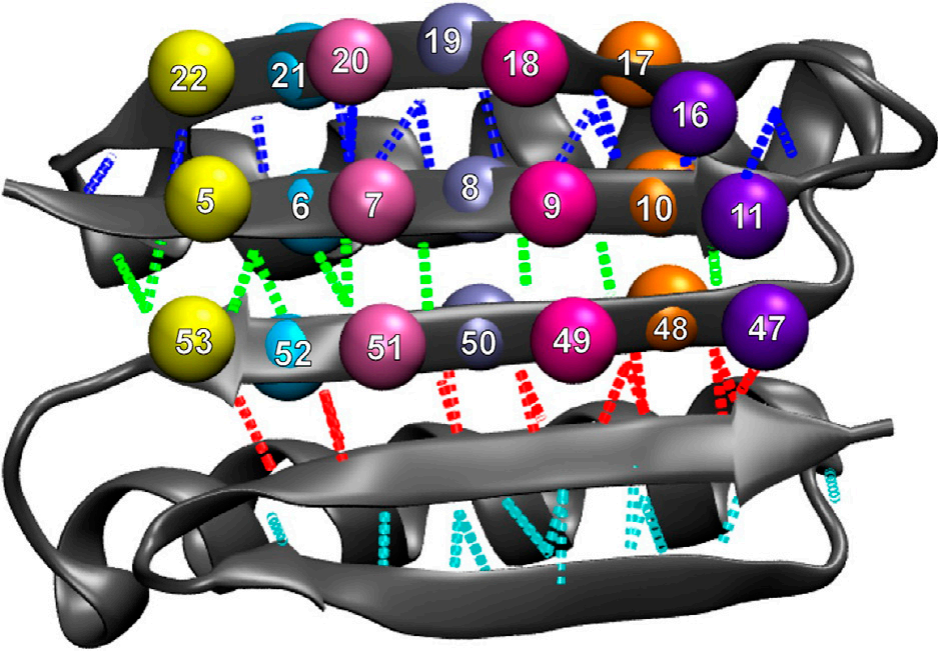
Residues involved in the first and second set (Figure 1) of hydrogen bonds and corresponding residue triplets used for the angle analysis (see Methods section). The colors of the hydrogen bonds correspond to those in Figure 1B, and the colors of triplets involving residues 16–19 correspond to those in Figure 6D (triplets are represented by identically colored Cα atoms).

We were particularly interested in the two edges of the beta-sheet, namely, residues 15–23 and 77–85, because these two beta strands are in direct contact with the terminal, force-bearing beta strands and are also exposed to water molecules. We started off by counting the total number of water molecules near strands 15–23 and 77–85 and the number of water molecules near specific residues (see Methods section). Figure 6 compares these values to the number of hydrogen bonds in the beta sheet. The number of water molecules near residues 15–23, particularly in residue 19, drops to its lowest level when the number of hydrogen bonds in the second set (Figure 1) falls to zero (Figures 6B,C). This result indicates that the second set of hydrogen bonds breaks at the moment when the side chain of residue Tyr-19, initially partially exposed to the solvent, gets buried. Tyrosine, although hydrophobic, has a polar side chain and is usually found on the surface of proteins. Notably, residue 19 is not directly implicated by our MI analysis in the rupture of contacts. However, in the neighboring strand (Figure 7), residue 9 is at the root of a structural disruption (residue 9 was detected regardless of the sliding window width *δ*, as shown in Figure 3). Disruptive neighbor residue 9 is bound to residue 49 via a hydrogen bond from the second set, which creates a viable pathway for destabilization. Additionally, a geometric analysis shows that the triplet angle formed by the Cα atoms of residue pairs 19/8 and 8/50 grows when the number of water molecules around the residue 19 drops (Figures 6, 7), indicating that the protein backbone near residue 19 moves with its side chain. This movement condenses residues 15–23 and thus forces the rotation of that segment, which is tightly bound to residues 5–11 by the first set of hydrogen bonds (Figures 1, 7). In turn, the compaction reinforces strands 15–23 and thus the first set of hydrogen bonds while increasing the distance between residues 8 and 50 and between residues 9 and 49, which are connected by the second set of hydrogen bonds (the distances between residues involved in the formation of the first set of hydrogen bonds remains stable; see Supplementary Figures S1B,C for more details). Thus, this mechanism breaks the second set of hydrogen bonds and enables the unfolding. The identical analyses performed on a slow-pulling simulation (1 m/s; Supplementary Figures S2, S3) and on another fast-pulling simulation (10 m/s; Supplementary Figures S4, S5) reveal very similar patterns, that is, the movement of residue 19 and its immediate tertiary neighbors are involved in the breakage of the second set of hydrogen bonds.

**FIGURE 8:**
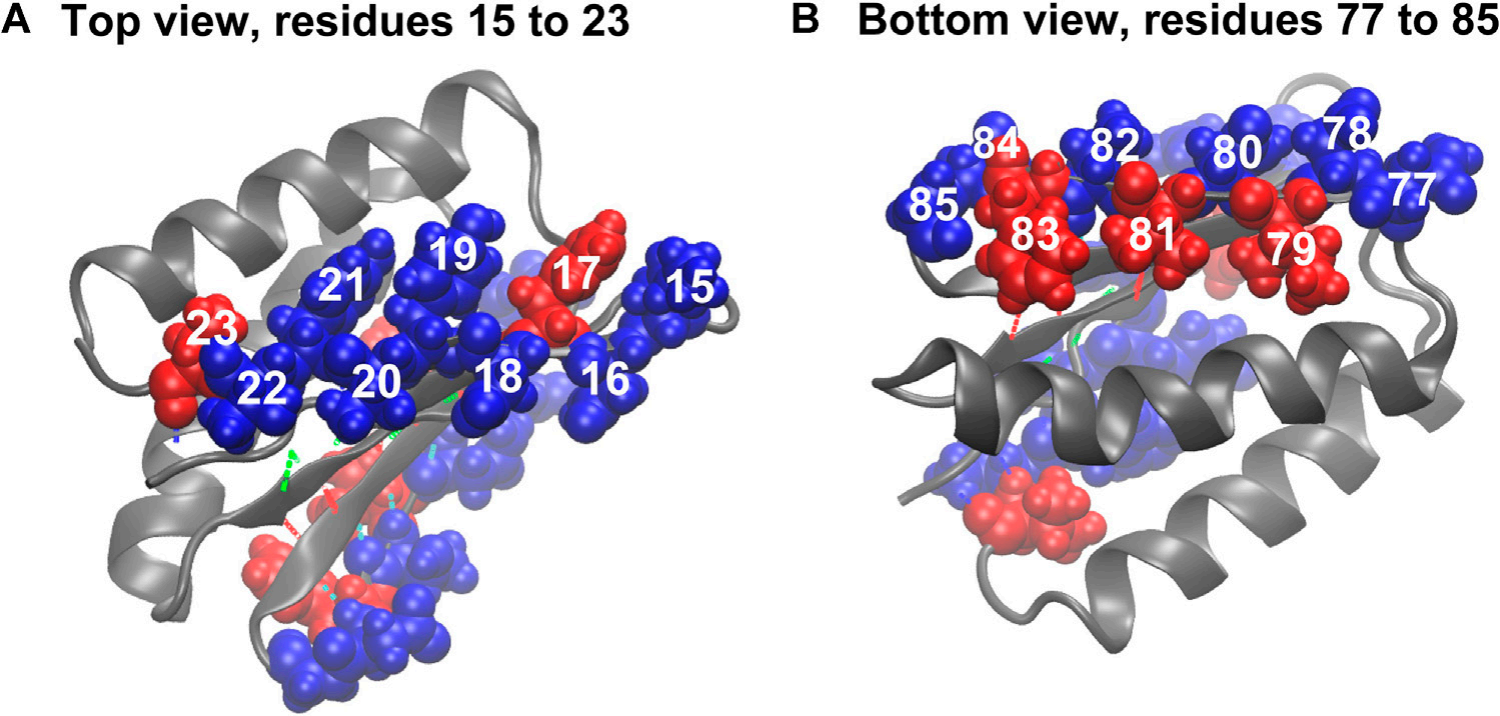
Beta sheet bordering hydrophobic (red) and hydrophilic (blue) residues. **(A)** Hydrophobic and hydrophilic residues in region I. **(B)** Hydrophobic and hydrophilic residues in region III. For hydrogen bond coloration, see Figure 1.

The comparison of the hydropathy patterns of residues 15–23 to residues 77–85 in Figure 8 shows that while residues 78–84 are alternatingly hydrophilic (78, 80, 82, and 84) and hydrophobic (79, 81, 83), residues 15–23 are mostly hydrophilic except for residues 17 and 23 (Figure 8). In the folded state, the sidechains of residues 15, 16, 18, 19, 20, 21, and 22 mostly prefer water molecules, whereas residue 17 (phenylalanine) tends to avoid the water environment (Figure 8). The side chain of residue 17 in equilibrium is oriented parallel to the side chains of residues 15 and 19. This finding indicates that due to the hydrophilicity of residues 15 and 19, residue 17 is also partially exposed to water in equilibrium, and a pronounced gap exists between the side chains of residues 17 and 19 (Figure 8A). This organization of residues is different from how residues 77–85 are organized in the symmetry-related region III, where the hydrophobic and hydrophilic side chains are highly regular, which does not put them in direct contact and allows a tighter packing of hydrophobic residues (79, 81, and 83). This finding explains why the third and fourth sets of hydrogen bonds (Figures 1B, 8) are relatively stable. The parallel orientation of the side chains of residues 15 (lysine, hydrophilic), 17 (phenylalanine, hydrophobic), and 19 (tyrosine, hydrophobic, but more soluble than phenylalanine) means that they can move more freely without experiencing steric clashes. The freedom of movement allows residues 17, 18, and 19 to react to mechanical stimuli and thus disrupt the second group of hydrogen bonds (Figures 1, 8) and the overall fold more readily.

In summary, by adding the hydropathy analysis and by measuring the concentration of water molecules around residues that break the hydropathy pattern, we can narrow our focus from residues 3–22 (after MI analysis) to strand 2 at the edge of the beta sheet. We observed a weak spot where side chains 16–22 have freedom of movement and thus would make suitable locations for the introduction of mutations intended to make the protein more mechanically stable. Such mutations would not introduce additional covalent bonds, as was the case with disulfide bridges proposed in earlier work (Sharma et al., 2007). Instead, we proposed to mutate one or more of these residues with hydrophilic side chains to residues with hydrophobic side chains (or vice versa) and bury them deeper into the body of the protein to mimic the hydropathy pattern in the stable region III (Figure 8B). Such a modification would pack them tight with the neighboring residues and restrict the possible movement of side chains during the perturbation, which should strengthen the weak spot.

## Discussion

Here, we presented an analysis of far from equilibrium SMD simulations of the Top7 protein by using MI statistics (Kovacs and Wriggers, 2016) to relate an external activity function or order parameter to the distance geometry of protein residues. The analysis evaluates how residue pair properties, such as the distance or its rate of change, correlate with an external activity function or order parameter along time. Thus, this analysis leads to a heat map of residue pairs that are likely to play an important part in the unfolding process. Earlier publications offer a detailed overview of the research leading to the development of MI protocols (Kovacs and Wriggers, 2016) and the adaptive probability density estimation algorithm (Kovacs et al., 2017). They also provide examples of the application of the protocols to MD simulations (Shaw et al., 2010; Kovacs and Wriggers, 2016; Wriggers et al., 2017). However, the present work was the first application of *TimeScapes* to SMD.

Our results show that the MI heatmap analysis of SMD is straightforward. The analysis based on the rate of change of the time series (absolute first derivative) emphasizes structural fluctuations of individual residues that are the result of force-induced hydrogen bond breaking (Figures 2, 3), whereas the direct MI analysis is based on the raw time-series predicted mechanically stable structural segments of the protein (Figure 4). The approaches provide complementary results that inform the subsequent analysis of the biophysical and biochemical properties of Top7. Specifically, the MI approaches select residues that belong to the beta sheets of region I (residues 3 to 22, in particular residues 9 and 12).

We also observed a modest dependence of the MI values on the duration *δ* used in the windowing of the activity function (Figures 2, 3). The statistics appear to be influenced by the time scale prescribed by the SMD pulling regime because the system is in a forced state of perturbation and orthogonal degrees of freedom are not sufficiently sampled. This finding is in contrast with prior equilibrium MD simulation applications, in which no dependence on *δ* was observed (Supplementary Figure S4 of Kovacs and Wriggers, 2016). In the SMD trajectory analysis, at the constant cutoff used for the selection in Figure 3, the smoothing parameter provides a trade-off between false negatives (only the strongest interactions are kept at large *δ*) and false positives (the analysis may include spurious correlations for small *δ*). The time-scale dependence of the peak MI values in the forced pulling simulations was modest and could easily be compensated for by an adjustment of the heuristic selection cutoff we employed after normalization.

Applications of the direct MI approaches to raw data (Figure 4) were originally developed for equilibrium simulations (Shaw et al., 2010) that exhibited pronounced fluctuations in the order parameter a(t) , which made them ideally suited for a statistical correlation analysis. In SMD, the system undergoes a near-linear directional change, so the RMSD in Figure 4A is shaped like a linear ramp function. Correlating it with pairwise distances in Figure 4B highlights the structural segments that are important for the directional change. We noted that for a simple ramp function, conventional analysis tools could have provided a heat map similar to Figure 4B (e.g., by plotting the pairwise residue distance changes between the start and the end conformation of the SMD run). However, a fluctuating order parameter, such as the RMSF in Figure 4C, justifies the use of the statistical MI analysis. In addition, the absolute time differential MI (Figure 2) is rooted in fluctuating activity functions a(t) .

The statistical dependence observed in Figure 4D between raw distances and the RMSF (which measures essentially the global rate of change of the system) is empirical, due to the absence of the absolute time derivatives (which in case of activity functions are conventionally applied to raw distances; Figure 2). Nevertheless, the unorthodox MI analysis of raw distances against RMSF (Figure 4D) picked up relevant interactions of helix I with its neighbors more clearly than the RMSD analysis (Figure 4B). Such intriguing “information fluidity” between order parameters that correspond to different degrees of time variation could be investigated further in future theoretical work.

The MI analysis does not prove causality, so we used conventional trajectory and hydropathy analyses to trim the initial MI predictions (residues 3–22) to a smaller subset (residues 16–22). The proposed rational design of more mechanically stable proteins is similar to other known experimental and modeling strategies aimed at engineering the hydrophobic effect (Ng et al., 2007; Sadler et al., 2009). Our results implicated two residues, 17 and 19, which in equilibrium have their sidechain more exposed to water (due to the opposed hydrophobic/hydrophilic characters of residues 17 and 19) in comparison to tightly packed residues in the region III of the protein. The exposure of the side chains of residues 17 and 19 to the solvent means that their mobility can influence the response of the protein to mechanical stimuli.

Our results also suggest that the hydrophobic effect, although being an equilibrium property, could drive the behavior of proteins in far-from-equilibrium regimes. This result opens a new avenue for the research of the rational design of mechanically stable proteins and molecules. The average hydropathy patterns largely follow the unfolding behavior of the Top7 protein, and the behavior of hydrophobic residues bordering the interface between regions I and II is largely concordant to the behavior of the second set (Figure 1) of beta-sheet hydrogen bonds. We do not claim that the hydrophobic effect directly influences mechanical behavior. The results only show how the protein equilibrium conformation (as influenced by the hydropathy patterns) could affect the folding process and the accessible unfolding path. This mechanism would be specific to Top7 because a relationship between the mechanical and thermodynamic properties has not yet been observed in other (evolutionarily designed) proteins (Bustamante et al., 2004).

Our work presents residues 16–22 as potential candidates for mutations that strengthen the protein. Those mutations should replace some of those residues (probably just one of them to preserve the global fold) with a more hydrophobic side chain that would be buried and anchored among neighboring residues. Another strategy would be to separate hydrophobic from hydrophilic side chains of residues 16–22, similar to the separation of hydrophobic from hydrophilic side chains in the stable region III. Therefore, the strategy may enable two unfolding pathways of similar resistance.

The residues with the highest score in the MI analysis in Figure 3A (residues 9 and 12) might provide targets for covalent cross-linking. However, such an intervention would be intrusive and restrict the unfolding path. (The residues are not ideal as potential mutant sites, given that residue 12 belongs to a loop and residue 9 is buried.) While these two residues are in the force-bearing strand 1, the residues were well connected to residues 16–22 in the neighboring strand through the first set of hydrogen bonds.

In summary, we propose a refined, non-covalent stabilization of residues 16–22 in strand 2 to rationally tune the mechanical resistance of Top7. To test the effect of any mutation on the protein folding and unfolding pathway, we can augment predictions by using fast perturbation experiments that detect both individual residues and rigid structural segments responsible for mechanical resistance. Our design approach can also form part of a “cocktail strategy” (Cao et al., 2011) that employs rather diverse modifications to rationally strengthen a protein.

## Data Availability

The analysis software presented in the study is documented and disseminated at http://timescapes.biomachina.org. Further inquiries can be directed to the corresponding authors.
