# Supplementary material for: Mechanism for the Unfolding of the TOP7 Protein in Steered Molecular Dynamics Simulations as Revealed by Mutual Information Analysis
Source: Front Mol Biosci. 2021 Sep 30;8:696609. doi: 10.3389/fmolb.2021.696609 (PMC8516001; doi:10.3389/fmolb.2021.696609)
Supplement: Supplementary file 1 [file Presentation1.pdf]

Supplementary Figures

Perišić and Wriggers, "Mechanism for the Unfolding of the TOP7 Protein in Steered Molecular Dynamics Simulations as Revealed by Mutual Information Analysis", Frontiers in Molecular Biosciences (2021)

Hydrogen bonds

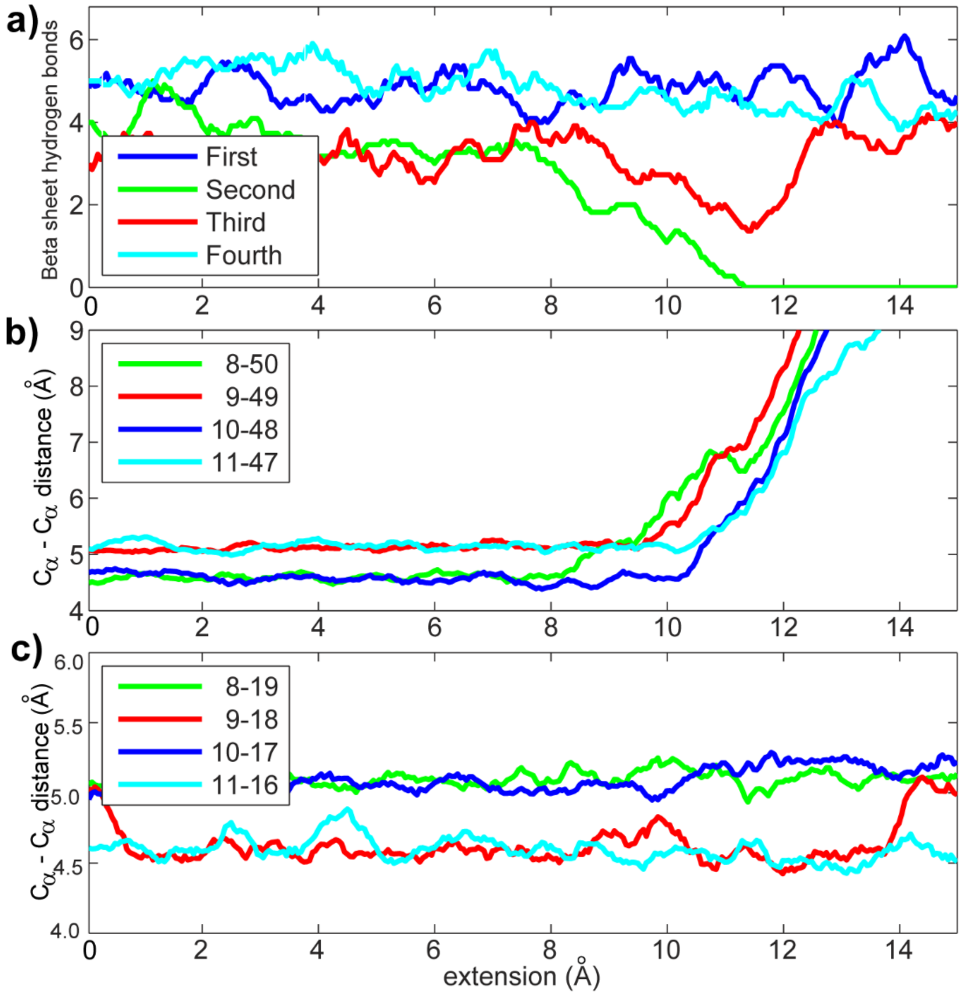

Supplementary Figure S1. Cα–Cα distances for residues involved in the formation of the first and second sets of beta-sheet hydrogen bonds for the fast-pulling simulation (10 m/s). a) Number of hydrogen bonds during the simulation for all four sets. The values are obtained through low-pass filtering (moving average with window width 11 frames, or +/- 5 frames about sampling points). b) Cα–Cα distances for the residues involved in the formation of the second set of hydrogen bonds. c) Cα–Cα distances for the residues involved in the formation of the first set of hydrogen bonds. All data values in Supplementary Figures S1–S5 are computed with VMD, as described in the Methods section of the main text.

Additional simulations (1 m/s and 10 m/s)

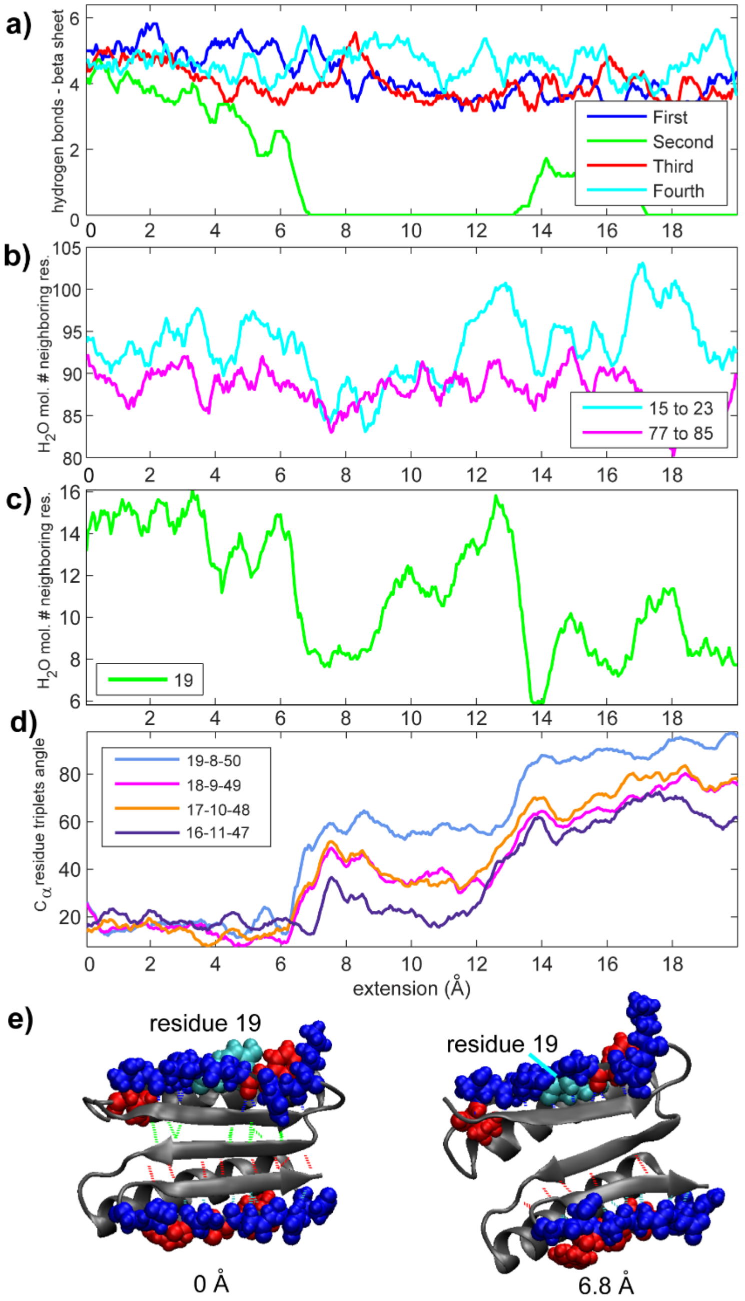

Supplementary Figure S2. Analyses of the numbers of hydrogen bonds vs. concentration of water molecules and dihedral angles for corresponding residues for the 1 m/s pulling trajectory (1 ns equilibration; 2.5 ns SMD length; max spring extension 25 Å; data analyzed as described in Fig. 6). a) Number of hydrogen bonds during the simulation. The values are obtained through low-pass filtering (moving average with window width 11 frames, or +/- 5 frames about sampling points). b) Numbers of water molecules near residues 15–23 and 77–85. c) Number of water molecules near residue 19. d) Dihedral angles for residue triplets 19–8–50, 18–9–49, 17–10–48, and 16–11–47. e) Structure of the protein during pulling. The molecular graphics in Supplementary Figures S2 and S4 were created with VMD, as described in the Methods section of the main text. Residue 19 (tyrosine is hydrophobic with polar side chain) is colored cyan. Hydrophobic residues (15–23 and 77–85) are colored red, and hydrophilic residues are colored blue (default VMD colors).

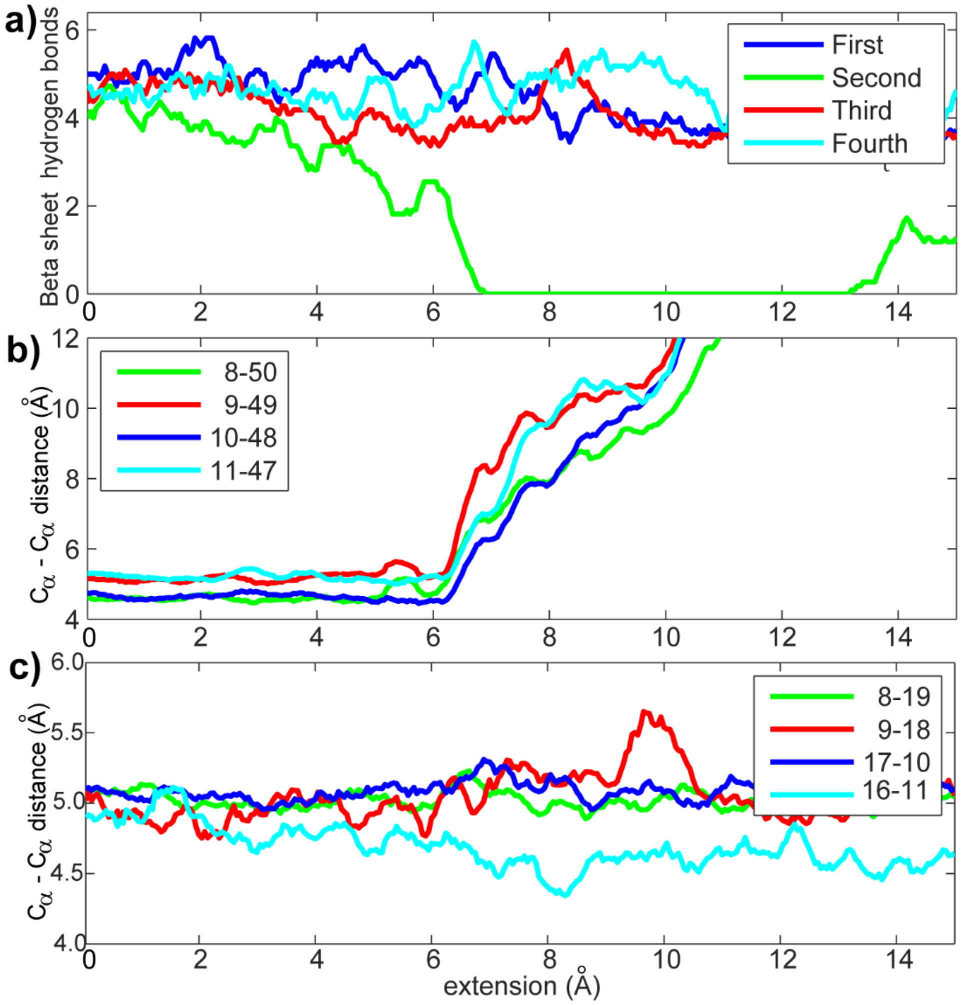

Supplementary Figure S3. Cα–Cα distances for residues involved in the formation of the first and second sets of beta-sheet hydrogen bonds for the slow-pulling simulation (1 m/s). a) Number of hydrogen bonds during the simulation for all four sets. The values are obtained through low-pass filtering (moving average with window width 11 frames, or +/- 5 frames about sampling points). b) Cα–Cα distances for the residues involved in the formation of the second set of hydrogen bonds. c) Cα–Cα distances for the residues involved in the formation of the first set of hydrogen bonds.

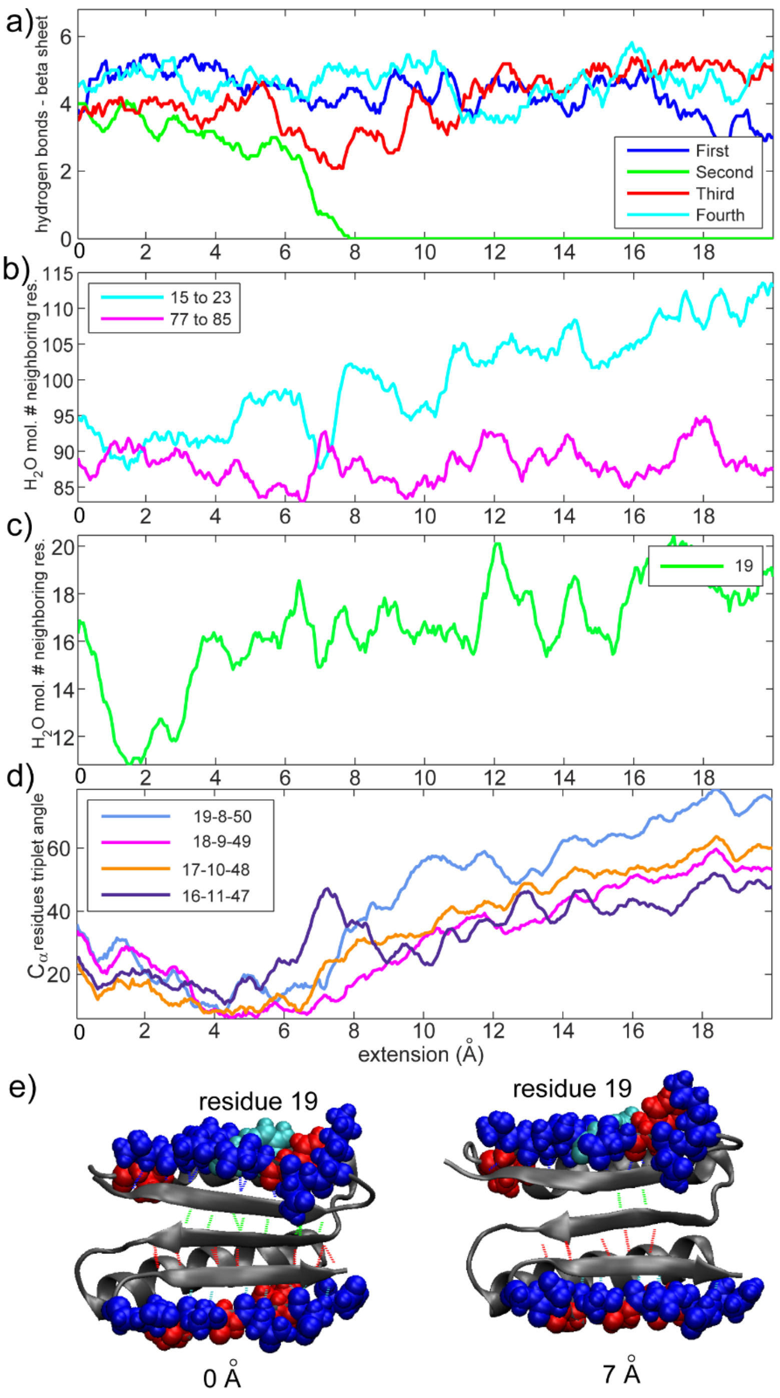

Supplementary Figure S4. Analyses of the numbers of hydrogen bonds vs. concentration of water molecules and dihedral angles for corresponding residues for the second 10 m/s pulling trajectory (1 ns equilibration as before + additional 0.05 ns equilibration to generate a different start structure; 0.5 ns SMD length; max spring extension 50 Å; data analyzed as described in Fig. 6). a) Number of hydrogen bonds during the simulation. The values are obtained through low-pass filtering (moving average with window width 11 frames, or +/- 5 frames about sampling points). b) Numbers of water molecules near residues 15–23 and 77–85. c) Number of water molecules near residue 19. d) Triplet angles for residue triplets 19–8–50, 18–9–49, 17–10–48, and 16–11–47. e) Structure of the protein during pulling. Residue 19 (tyrosine is hydrophobic with polar side chain) is colored cyan. Hydrophobic residues (15–23 and 77–85) are colored red, and hydrophilic residues are colored blue (default VMD colors). In this second 10 m/s simulation, the breaking of the second set of hydrogen bonds in

a), and the drop in residue 19 solvation in c), occurred earlier than in the first simulation (Fig. 6), although the unfolding process was consistent.

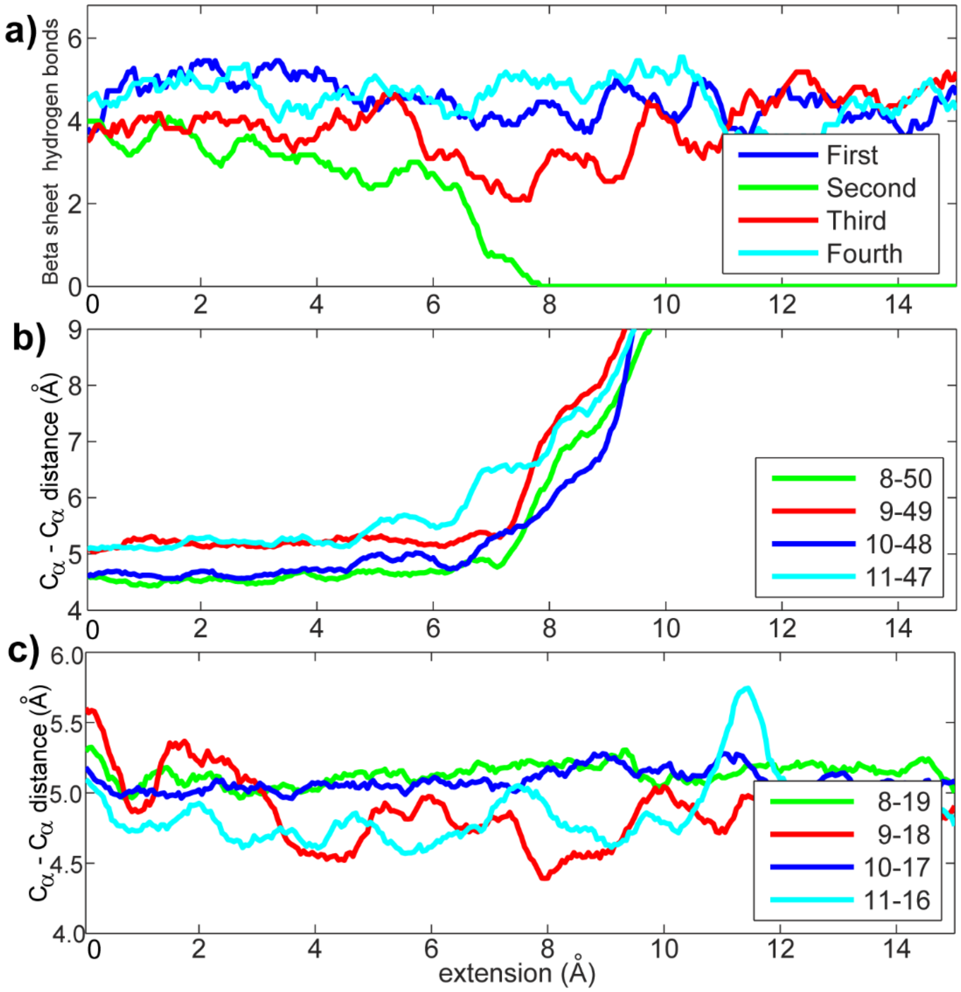

Supplementary Figure S5. Cα – Cα distances for residues involved in the formation of the first and second sets of beta-sheet hydrogen bonds for the second fast-pulling simulation (10 m/s). a) Number of hydrogen bonds during the simulation for all four sets. The values are obtained through low-pass filtering (moving average with window width 11 frames, or +/- 5 frames about sampling points). b) Cα–Cα distances for the residues involved in the formation of the second set of hydrogen bonds. c) Cα–Cα distances for the residues involved in the formation of the first set of hydrogen bonds.
